# **β** Cell function after Roux-en-Y gastric bypass surgery or reduced energy intake alone in people with obesity

**DOI:** 10.1172/jci.insight.170307

**Published:** 2023-06-22

**Authors:** Bettina Mittendorfer, Bruce W. Patterson, Faidon Magkos, Mihoko Yoshino, David P. Bradley, J. Christopher Eagon, Samuel Klein

**Affiliations:** Center for Human Nutrition, Washington University School of Medicine, St. Louis, Missouri, USA.

**Keywords:** Metabolism, Beta cells, Insulin, Obesity

## Abstract

**Background:**

The effects of diet-induced weight loss (WL) and WL after Roux-en-Y gastric bypass (RYGB) surgery on β cell function (BCF) are unclear because of conflicting results from different studies, presumably because of differences in the methods used to measure BCF, the amount of WL between treatment groups, and baseline BCF. We evaluated the effect of WL after RYGB surgery or reduced energy intake alone on BCF in people with obesity with and without type 2 diabetes.

**Methods:**

BCF (insulin secretion in relationship to plasma glucose) was assessed before and after glucose or mixed-meal ingestion before and after (a) progressive amounts (6%, 11%, 16%) of WL induced by a low-calorie diet (LCD) in people with obesity without diabetes, (b) ~20% WL after RYGB surgery or laparoscopic adjustable gastric banding (LAGB) in people with obesity without diabetes, and (c) ~20% WL after RYGB surgery or LCD alone in people with obesity and diabetes.

**Results:**

Diet-induced progressive WL in people without diabetes progressively decreased BCF. Marked WL after LAGB or RYGB in people without diabetes did not alter BCF. Marked WL after LCD or RYGB in people with diabetes markedly increased BCF, without a difference between groups.

**Conclusion:**

Marked WL increases BCF in people with obesity and diabetes but not in people with obesity without diabetes. The effect of RYGB-induced WL on BCF is not different from the effect of matched WL after LAGB or LCD alone.

**trial registration:**

NCT00981500, NCT02207777, NCT01299519.

**Funding:**

NIH grants R01 DK037948, P30 DK056341, P30 DK020579, UL1 TR002345.

## Introduction

Insulin resistance is the most common metabolic abnormality associated with obesity. Therefore, adequate basal and postprandial insulin secretion from pancreatic β cells is particularly important in people with obesity to prevent prediabetes and type 2 diabetes (T2D), which are caused by insulin resistance and impaired β cell function (defined as insulin secretion in relationship to plasma glucose) (1, 2). In fact, the severity of β cell dysfunction is the major determinant of developing T2D rather than prediabetes because insulin resistance is often not different between people with prediabetes and T2D (2, 3). It has been proposed that weight loss improves β cell function and that weight loss after Roux-en-Y gastric bypass (RYGB) surgery has additional beneficial effects on β cell function compared with weight loss alone because RYGB causes a greater increase in postprandial plasma glucagon-like peptide 1 (GLP-1) and gastric inhibitory peptide (GIP) concentrations that enhance glucose-stimulated insulin secretion (4, 5). However, the effects of diet-induced weight loss and RYGB surgery–induced weight loss on β cell function are unclear because of conflicting results from different studies (4–13). A recent meta-analysis (13) concluded that it is not feasible to draw firm conclusions on the effect of weight loss on β cell function due to the heterogeneity in experimental designs among studies that includes differences in the methods used to measure β cell function, differences in the amount of weight loss between study groups, and differences in the presence or absence of T2D in study participants and, therefore, differences in β cell function at baseline.

The ability of pancreatic β cells to secrete adequate amounts of insulin in relationship to plasma glucose during both fasting and postprandial conditions involves a complex series of events that integrates nutritional, hormonal, and neural factors (14, 15). Many different methods have been used to evaluate the effects of weight loss, induced by diet alone or by bariatric surgery, on β cell function, including basal insulin secretion rate (ISR), the change in ISR and insulin concentration in response to a dynamic metabolic perturbation induced by glucose or meal ingestion, i.v. glucose bolus injection or continuous infusion, or i.v. arginine infusion (4–13, 16–18). However, plasma insulin concentration does not provide a reliable assessment of insulin secretion because it does not take into account potential differences in plasma insulin clearance between groups or the change in plasma insulin clearance that can occur with weight loss (19, 20). In addition, providing an i.v. glucose stimulus does not mimic the normal route and pattern of glucose delivery that occurs after glucose or meal ingestion and does not elicit a gastrointestinal incretin or neural response (14, 21). This limitation is a particular concern in evaluating β cell function after RYGB surgery because of the profound effect of surgery on the rate that ingested glucose is absorbed and delivered into the systemic circulation (8, 9). Therefore, an assessment of ISR in relation to increasing plasma glucose concentrations before and after glucose or mixed-meal ingestion provides the most clinically relevant assessment of β cell function because it involves all of the physiological factors that regulate insulin release from β cells.

The purpose of this study was to evaluate the effect of weight loss induced by RYGB surgery or reduced energy intake alone on β cell function in people with obesity with and without T2D. Specifically, we assessed (a) the effect of progressive amounts (6%, 11%, and 16%) of weight loss (progressive weight loss [PWL]) induced by a low-calorie diet (LCD), compared with weight maintenance (WM), on β cell function in people with obesity without diabetes (OB-PWL and OB-WM groups); (b) the effect of marked (~20%) weight loss induced by laparoscopic adjustable gastric banding (LAGB) or RYGB surgery on β cell function in people with obesity without diabetes (OB-LAGB and OB-RYGB groups); and (c) the effect of marked (~20%) weight loss induced by a LCD alone or RYGB surgery in people with obesity and T2D (T2D-LCD and T2D-RYGB groups). β Cell function was assessed as the relationship between ISR and plasma glucose concentration during basal conditions and during the early postprandial period (after glucose ingestion in the OB-PWL and OB-WM groups and after mixed-meal ingestion in the OB-LAGB, OB-RYGB, T2D-LCD, and T2D-RYGB groups) when plasma glucose concentration is rapidly rising and provides the greatest challenge to β cells. We hypothesized that weight loss would improve β cell function (i.e., increase in ISR in relation to plasma glucose) in people with T2D but would have minimal or no effect on β cell function in those without T2D. Furthermore, we hypothesized that marked weight loss (~20% decrease in body weight) induced by RYGB surgery would cause a greater increase in β cell function than matched weight loss induced by LAGB or LCD therapy alone.

## Results

### Age, body composition, and insulin sensitivity before and after weight loss.

Age, baseline body weight and composition, and glucose-related metabolic variables were not different between the OB-PWL and OB-WM groups in Study 1-PWL (see Table 1 and Methods). Age and baseline body weight and composition were also not different between the OB-LAGB and OB-RYGB groups in Study 2-LAGB versus RYGB (see Methods) and the T2D-LCD and T2D-RYGB groups in Study 3-LCD versus RYGB (see Methods) (Table 2). PWL in participants in the OB-PWL group caused a marked increase in insulin-stimulated glucose disposal rate that plateaued at 11% weight loss (Table 1). The increases in insulin-stimulated glucose disposal rate induced by 20% weight loss were not different between the OB-LAGB and OB-RYGB groups or between the T2D-LCD and T2D-RYGB groups (Table 2).

### Effect of PWL on plasma glucose and insulin concentrations and insulin kinetics in participants without T2D.

**Figure 1:**
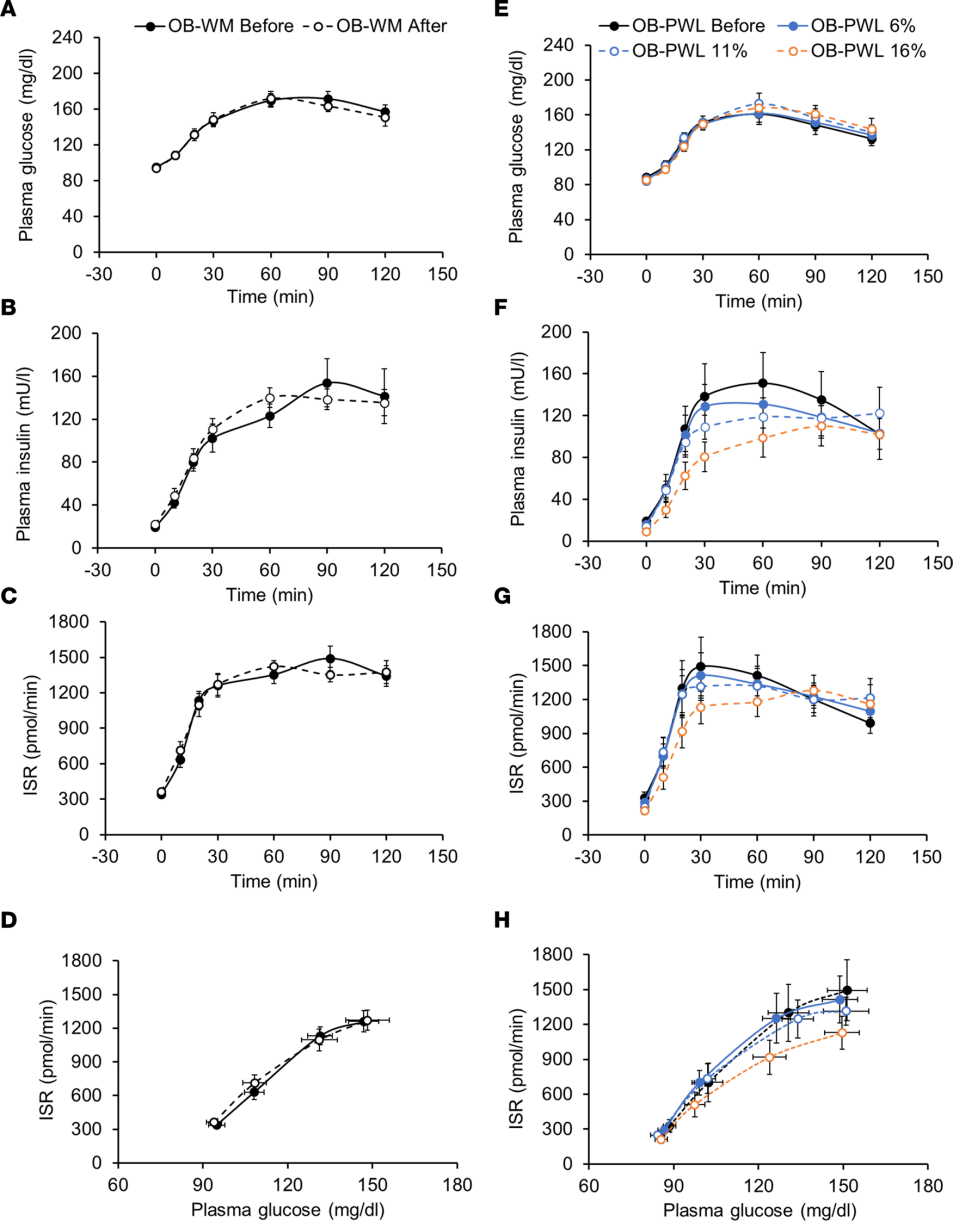
Metabolic response to ingesting 75 grams of glucose before and after weight maintenance or progressive weight loss induced by a low-calorie diet in people with obesity without type 2 diabetes. (**A**–**C** and **E**–**G**) Plasma glucose (**A** and **E**) and insulin (**B** and **F**) concentrations, and insulin secretion rate (**C** and **G**) before and after ingesting 75 grams of glucose. (**D** and **H**) The relationship between insulin secretion rate and plasma glucose concentration during the early postprandial period (first 30 min) after glucose ingestion when plasma glucose was rapidly rising before and after weight maintenance (left, **A**–**D**) and before and after progressive 6%, 11%, and 16% weight loss induced by a low-calorie diet (right, **E**–**H**). Data are expressed as mean ± SEM. OB-PWL, *n* = 9; OB-WM, *n* = 14. ISR, insulin secretion rate; OB, obese; PWL, progressive weight loss; WM, weight maintenance.

Fasting plasma glucose concentration and plasma glucose concentrations for 120 minutes after glucose ingestion were not different after 6%, 11%, and 16% weight loss compared with baseline in the OB-PWL group and before and after WM in the OB-WM group (Table 1 and Figure 1, A and E). However, fasting plasma insulin and C-peptide concentrations, basal ISR, and plasma insulin concentration and ISR after glucose ingestion decreased progressively with progressive amounts of weight loss (Table 1 and Figure 1, F and G) and did not change in the OB-WM group (Table 1 and Figure 1, B and C). Plasma C-peptide concentration and ISR in relation to plasma glucose concentration during basal conditions and during the first 30 minutes after glucose ingestion, when plasma glucose was rising, progressively decreased with progressive amounts of weight loss (linear trend, *P* < 0.05) and was significantly lower after 16% weight loss compared with baseline (*P* < 0.05) (Figure 1H and Supplemental Figure 1; supplemental material available online with this article; https://doi.org/10.1172/jci.insight.170307DS1). In the OB-WM group, β cell function assessed after approximately 6 months was not different from β cell function at baseline (Figure 1D).

### Effect of marked weight loss after LAGB or RYGB on plasma glucose and insulin concentrations and on insulin kinetics in participants without T2D.

**Figure 2:**
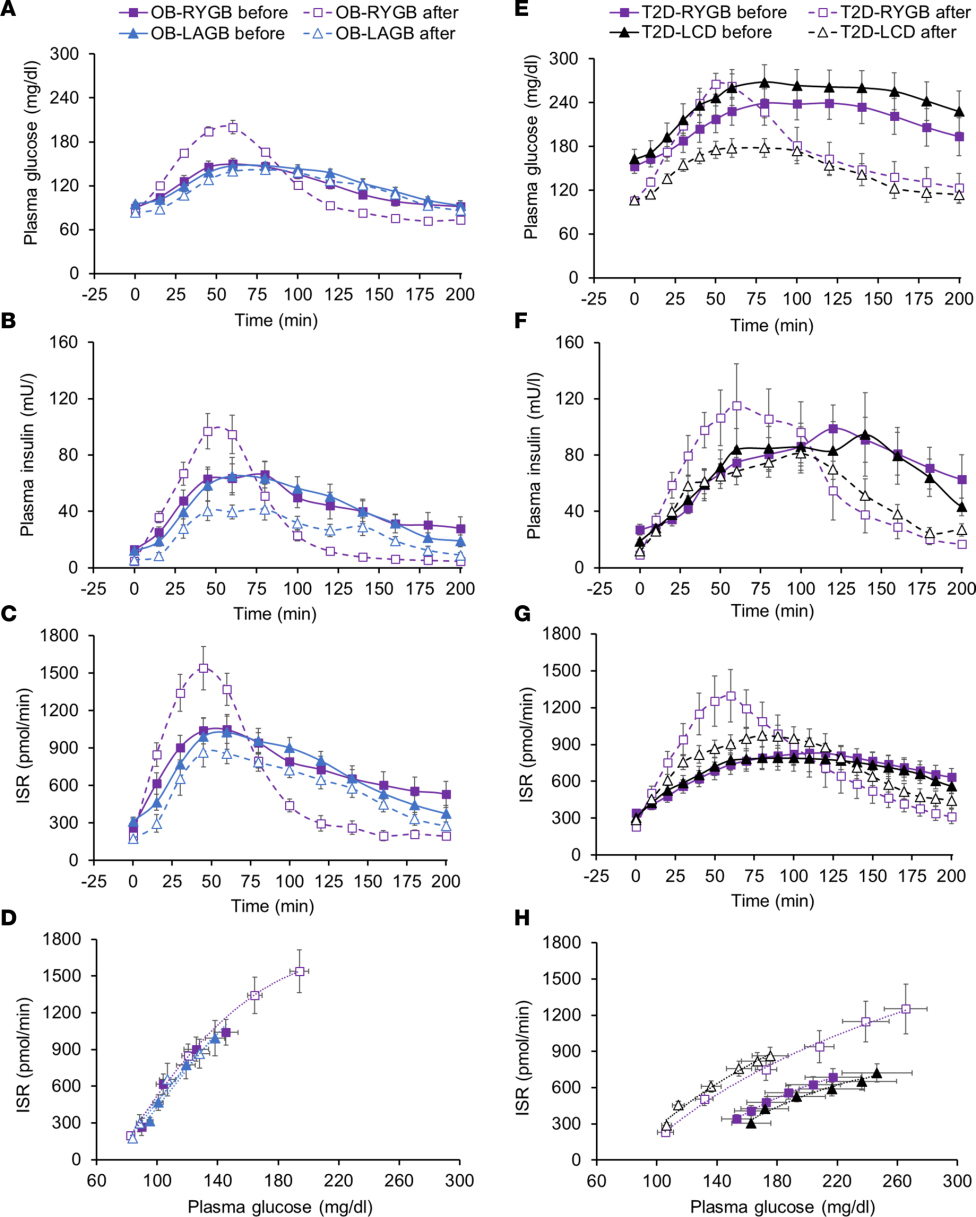
Metabolic response to ingesting a mixed meal containing ~50 g of glucose before and after marked (~20%) weight loss induced by a low-calorie diet alone or laparoscopic gastric banding or Roux-en-Y gastric bypass surgery. (**A**–**D**) Basal and postprandial plasma glucose (**A**) and insulin (**B**) concentrations, insulin secretion rate (**C**), and the relationship between insulin secretion rate and plasma glucose concentration during the early postprandial period (first 45 min after initiating liquid mixed meal ingestion) when plasma glucose was rapidly rising (**D**) before and after weight loss induced by Roux-en-Y gastric bypass surgery or laparoscopic gastric banding. (**E**–**H**) Basal and postprandial plasma glucose (**E**) and insulin (**F**) concentrations, insulin secretion rate (**G**), and the relationship between insulin secretion rate and plasma glucose concentration during the early postprandial period (first 50 min after initiating liquid mixed meal ingestion) when plasma glucose was rapidly rising (**H**) before and after weight loss induced by Roux-en-Y gastric bypass surgery or a low-calorie diet alone in people with obesity and type 2 diabetes. ISR, insulin secretion rate; LAGB, laparoscopic adjustable gastric banding; LCD, low-calorie diet; OB, obese; RYGB, Roux-en-Y gastric bypass; T2D, type 2 diabetes. Data are expressed as mean ± SEM. OB-LAGB, *n* = 11; OB-RYGB, *n* = 12; T2D-LCD, *n* = 10; T2D-RYGB, *n* = 9.

Fasting plasma glucose, insulin and C-peptide concentrations, and basal ISR were lower after than before weight loss in both the OB-LAGB and OB-RYGB groups without differences between groups (Table 2). Compared with weight loss after LAGB, weight loss after RYGB surgery caused an earlier and initially (during the first hour) greater postprandial increase in plasma glucose and insulin concentrations (Figures 2, A and B). The plasma glucose concentration AUC during the 200-minute postprandial period (AUC_0–200_) was not different before and after weight loss in either the OB-LAGB group or the OB-RYGB group (Table 2). The plasma insulin concentration AUC_0–200_ was much lower (~30%) after than before weight loss in both the OB-LAGB group and the OB-RYGB group (Table 2), without a difference between groups (main effect of time, *P* < 0.05; no significant group × time interaction). The ISR during the first hour after mixed-meal ingestion was much greater after than before weight loss in the OB-RYGB group and greater in the OB-RYGB than the OB-LAGB group after weight loss, but it declined rapidly thereafter to values below those in the OB-LAGB group (Figure 2C). The ISR AUC_0–200_ was about 15% lower after than before weight loss in both the OB-LAGB group and the OB-RYGB group (Table 2), without a difference between groups (main effect of time, *P* < 0.05; no significant group × time interaction). The relationship between ISR and plasma glucose concentration during basal conditions and during the early postprandial period when plasma glucose was rising was not different between the OB-LAGB and OB-RYGB groups at baseline and was not altered by weight loss in either group (Figure 2D).

### Effect of marked weight loss induced by a LCD alone or RYGB on plasma glucose and insulin concentrations and on insulin kinetics in participants with T2D.

Weight loss after both the LCD intervention and RYGB surgery caused a marked (~35%) decrease in both basal plasma glucose concentration and plasma glucose concentration AUC_0–200_, without a difference between the T2D-LCD and the T2D-RYGB groups (Table 2 and Figure 2E). Basal plasma insulin and C-peptide concentrations and ISR were lower after than before weight loss in both the T2D-LCD and T2D-RYGB groups, without a difference between the 2 groups (Table 2). The increases in plasma glucose and insulin concentrations within the first 60 minutes after mixed-meal ingestion were much greater after weight loss in the T2D-RYGB than the T2D-LCD group (Figure 2, E and F). However, plasma glucose concentration AUC_0–200_ decreased after weight loss in both the T2D-RYGB group and the T2D-LCD group, without a difference between the 2 groups, whereas weight loss did not affect plasma insulin concentration AUC_0–200_ in either the T2D-RYGB or the T2D-LCD groups (Table 2 and Figure 2F). The increase in ISR during the first hour after mixed-meal ingestion was much greater after weight loss in the T2D-RYGB than the T2D-LCD group (Figure 2G), but the ISR AUC_0–200_ was not different before and after weight loss in either the T2D-LCD group or the T2D-RYGB group (Table 2).

Before weight loss, the relationship between ISR and plasma glucose concentration during basal conditions and during the early postprandial period, when plasma glucose was rising, was not different between the T2D-LCD and T2D-RYGB groups (Figure 2H). The ISR at any plasma glucose concentration was greater after than before weight loss in both the T2D-LCD and T2D-RYGB groups (*P* < 0.01), without a difference between the 2 groups (Figure 2H). However, the ISR in the groups with T2D was still much lower than in participants without diabetes (Figures 2, D and H), demonstrating that β cell function was still markedly impaired after weight loss in the groups with T2D.

## Discussion

In the present study, we evaluated the effect of weight loss and concomitant improvements in whole-body insulin sensitivity, induced by diet therapy alone and by bariatric surgical procedures that either maintain (LAGB) or disrupt the continuity of the upper gastrointestinal tract (RYGB) on β cell function in people with obesity with and without T2D. β Cell function was assessed by determining the ISR in relation to plasma glucose concentration during basal conditions and early after glucose or mixed-meal ingestion, when plasma glucose concentration is rapidly increasing. This assessment provides an evaluation of normal physiological β cell dynamics that involve the direct effect of glucose on β cells and the amplifying effect of the glucose ingestion–mediated secretion of intestinal incretins that bind to GLP-1 and GIP receptors on β cells and augment glucose-stimulated insulin secretion (14, 21, 22). Our data demonstrate that (a) PWL (6%, 11%, 16%), induced by a LCD, in people with obesity without diabetes causes a progressive decrease in insulin secretion in relation to plasma glucose; (b) marked (~20%) weight loss, induced by LAGB and RYGB, in people with obesity without diabetes does not alter insulin secretion in relation to plasma glucose; and (c) marked (~20%) weight loss, induced by a LCD alone or RYGB, causes a marked increase in insulin secretion in relation to plasma glucose, without a difference between the T2D-LCD and T2D-RYGB groups. Although both the OB-RYGB and T2D-RYGB groups demonstrated a much greater early postprandial increase in insulin secretion than the OB-LAGB and T2D-LCD groups, the ISR in relation to plasma glucose was not different between the participants who had RYGB and those who had lost the same amount of weight after LAGB or a LCD alone. These data demonstrate that marked weight loss increases β cell function in people with obesity and diabetes (i.e., those with marked β cell dysfunction), and this contributes to robust improvements in basal and postprandial glycemia. However, marked weight loss does not affect β cell function or even decreases it in people with obesity without diabetes, who typically have normal or increased β cell function (2). Nevertheless, basal and postprandial plasma glucose concentrations do not change or even decrease after weight loss in people without T2D, because of the weight loss–induced increase in insulin sensitivity, which causes greater insulin action for a given amount of plasma insulin.

Insulin secretion from pancreatic β cells represents a complex and coordinated cellular response that is initiated by β cell glucose oxidation, which causes the closure of ATP-sensitive potassium (K_ATP_) channels, resulting in an increase in cytosolic calcium that triggers a series of downstream events that cause exocytosis of insulin-containing granules (14). Healthy β cells respond to glucose uptake and oxidation by secreting the amount of insulin needed to maintain “normal” basal and postprandial plasma glucose concentrations, whereas inadequate insulin secretion causes “abnormally high” plasma glucose. Therefore, less insulin secretion is required to maintain normoglycemia in people who are insulin sensitive compared wit those who are insulin resistant. The mechanisms responsible for the different effects of weight loss on β cell function in people with and without T2D are likely related to differences in β cell function between groups at baseline (before weight loss). β Cell function was much worse in our participants with T2D (T2D-LCD and T2D-RYGB) than in those without T2D (OB-PWL, OB-WM, OB-LAGB, and OB-RYGB), presumably because of decreased β cell mass (23–25), altered β cell ultrastructure (26), and disordered β cell electrical activity (27, 28). The results from our study are consistent with the proposed paradoxical effect of β cell K_ATP_ current density on glucose-stimulated insulin secretion (27, 28). A decrease in K_ATP_ current density, due to glucose-mediated K_ATP_ channel closure, in conjunction with decreased K_ATP_ channel recruitment to the plasma membrane associated with obesity increases glucose-stimulated insulin secretion, whereas a marked decrease in K_ATP_ channel activity below a certain threshold decreases glucose-stimulated insulin secretion (27, 28). This “crossover effect” of K_ATP_ current density on insulin secretion likely contributes to the progressive decline in β cell function that occurs as people progress from obesity and normal glycemic control (insulin hypersecretion) to obesity and prediabetes (insufficient insulin secretion) or to obesity and T2D (markedly impaired insulin secretion) (2, 27, 28). According to this paradigm, weight loss increases K_ATP_ current density, which increases glucose-stimulated insulin secretion in people with T2D who have “crossed over” but decreases glucose-stimulated insulin secretion in people with obesity who have not “crossed over,” as observed in our participants. In addition, it is likely that increases in β cell mass and remodeling of β cell ultrastructure also contributed to the improvement in β cell function in the T2D-LCD and T2D-RYGB groups. Studies conducted in obese diabetic rats found that (a) weight loss increases β cell number and total β cell mass (29), and (b) even a small (11 mg/dL) chronic increase in plasma glucose concentration has adverse effects on the expression of islet cell genes associated with β cell viability and function (30), whereas decreasing plasma glucose with pharmacotherapy improves β cell ultrastructure and function, independently of weight loss (31). Although the results from our study appear to contradict previous studies that concluded that weight loss increases β cell function in people with obesity without T2D (8, 18, 20, 32, 33), β cell function in those studies was evaluated by using the disposition index or other indices that assessed insulin secretion/insulin concentration in relation to insulin sensitivity (34, 35). The improvement in “β cell function” in these studies was primarily due to increased insulin sensitivity, not increased insulin secretion in relation to plasma glucose (8, 18, 20, 32, 33).

Weight loss induced by RYGB surgery causes a much greater increase in postprandial plasma incretins (GLP-1 and GIP) than weight loss induced by LAGB or a LCD alone (4, 5). It has been proposed that the enhanced incretin release associated with RYGB improves β cell function and glycemic control (4, 5, 36, 37). Our data show that β cell function (i.e., insulin secretion in relation to plasma glucose) after RYGB is appropriate for the rapid delivery of ingested glucose into the systemic circulation. In fact, the effect of weight loss induced by RYGB on β cell function was identical to the effect of weight loss induced by LAGB or a LCD alone in participants with and without T2D. Our findings are consistent with the results from previous studies that found marked weight loss after RYGB improved β cell function, assessed as glucose-stimulated insulin secretion in response to a mixed-meal test, a graded glucose infusion protocol, or a hyperglycemic clamp procedure, in people with T2D but did not improve or even decreased β cell function 1–2 years after marked weight loss induced by RYGB in people without diabetes (6, 7, 16, 33). These data demonstrate that (a) the increase in postprandial incretin secretion after RYGB is a normal response to the altered intestinal delivery of glucose but does not enhance β cell function more than expected in relation to the postprandial rise in plasma glucose and (b) the effect of weight loss on β cell function is different in people with and without T2D.

Our study has several strengths, including the inclusion of participants with and without T2D, the matched weight loss in the RYGB and comparator groups, and the assessment of β cell function during progressive amounts of weight loss. In addition, we evaluated β cell function during basal conditions and after glucose or mixed-meal ingestion, which has greater clinical relevance than assessing the β cell response to i.v. glucose infusion. Nevertheless, our study is also limited because the data represent a secondary analysis of data from 3 different studies and the 2 surgery studies were not randomized controlled trials. However, the metabolic characteristics of the participants, which are critical in the assessment of outcomes across study groups, were rigorously evaluated, and the methods used to assess the key outcome measures (ISR and plasma glucose concentration) were identical across studies. Furthermore, repeat testing was conducted about 6–8 weeks sooner after RYGB surgery than LAGB surgery and LCD alone because of the more rapid weight loss after RYGB than LAGB and LCD alone. In addition, most of our study participants were women, so we are unable to determine whether there are sex differences in the effects of diet and bariatric surgery–induced weight loss on metabolic outcomes.

In summary, the data from our study demonstrate that marked weight loss improves β cell function, defined as insulin secretion in relation to plasma glucose, in people with obesity and T2D, and the effect of weight loss induced by RYGB surgery on β cell function is not different from the effect of matched weight loss induced by a LCD alone. In contrast, marked weight loss induced by RYGB, LAGB, or diet alone does not improve, or even decreases, β cell function, in people with obesity who do not have T2D.

## Methods

The data in this manuscript represent a secondary analysis of data obtained from 3 previously published prospective clinical trials (8, 9, 20) and data obtained from additional participants who fulfilled the same inclusion criteria and completed the same study protocols (ClinicalTrials.gov; NCT00981500, NCT02207777, and NCT01299519) that evaluated insulin sensitivity (glucose disposal rate during a hyperinsulinemic-euglycemic clamp procedure) and the metabolic response to an oral glucose tolerance test or mixed-meal ingestion before and after weight loss but have not been published. Data from participants who were enrolled in the following studies were analyzed: (a) Study 1-PWL, conducted in participants with obesity without diabetes who were randomized to either a progressive (6%, 11%, 16%) weight loss intervention (OB-PWL group) or WM (OB-WM group); (b) Study 2-LAGB versus RYGB, conducted in participants with obesity without diabetes who were studied before and after ~20% weight loss induced by LAGB (OB-LAGB group) or RYGB (OB-RYGB group); and (c) Study 3-LCD versus RYGB, conducted in participants with obesity and T2D who were studied before and after approximately 20% weight loss induced by a LCD alone (T2D-LCD group) or RYGB surgery (T2D-RYGB group). Detailed descriptions of participant selection criteria and the study protocols are provided in the relevant publications (8, 9, 20).

### Study participants.

All participants completed a screening evaluation after they fasted for 12 hours overnight; the evaluation included a medical history and physical examination as well as standard blood tests. Potential participants were excluded if they (a) had a disease (other than T2D) or were taking any medication that could affect the study outcome measures; (b) had previous bariatric surgery; or (c) consumed excessive amounts of alcohol (men, >21 drinks per week; women, >14 drinks per week). Participants with T2D were instructed to stop taking GLP-1 receptor agonists for 2 weeks, oral diabetes medications for 3 days, and insulin for 1 day before each admission to the Clinical and Translational Research Unit at Washington University School of Medicine for metabolic testing.

### Body composition analysis and metabolic testing.

Body fat mass and fat-free mass were determined by using dual energy X-ray absorptiometry (Lunar iDXA, GE Healthcare Lunar). Insulin sensitivity was assessed as insulin-stimulated glucose disposal rate in relation to fat-free mass during a hyperinsulinemic-euglycemic clamp procedure (insulin infusion rate: 50 mU/m^2^ body surface area/min) in conjunction with [6,6-^2^H_2_]glucose infusion (8, 9, 20). Plasma glucose, insulin, and C-peptide concentrations were assessed before and at specific times for 120 minutes after participants consumed 75 g of glucose (OB-PWL and OB-WM groups) and before and at specific times for 200 minutes after participants consumed a liquid mixed meal that contained approximately 50 g of glucose (OB-LAGB, OB-RYGB, T2D-LCD, and T2D-RYGB groups). Blood samples were obtained in 10- to 15-minute intervals for the first hour and thereafter in 20- to 30-minute intervals, as previously described (8, 9, 20). ISR was determined by fitting the plasma C-peptide concentrations at each time point to a 2-compartment model as previously described (38).

### Bariatric surgery and weight loss interventions.

In Study 1-PWL, participants were randomly assigned to treatment with a LCD or WM. After participants achieved each weight loss target (6%, 11%, and 16%), a WM diet was prescribed to maintain a stable body weight (<2% change) for 3 weeks before repeat testing was performed. The time interval between baseline testing and follow-up testing was about 6 months in the OB-WM group and about 1 year after 16% weight loss in the OB-PWL group.

In Study 2-LAGB versus RYGB and Study 3-LCD versus RYGB, the surgery procedures were performed by using standard techniques within a few weeks after baseline testing was completed. All participants (LCD and surgery groups) in both studies participated in a supervised dietary weight loss program designed to achieve 20% weight loss followed by a weight-maintenance diet to maintain a stable body weight (<2% change) for at least 2 weeks before the metabolic assessments were repeated. On average, repeat testing was conducted about 4–5 months after LAGB and RYGB surgery (22 ± 2 weeks in the LAGB group; 15 ± 1 weeks in the OB-RYGB group; 16 ± 1 weeks in the T2D-RYGB group) and within about 6 months (23 ± 2 weeks) after starting the diet intervention in the T2D-LCD group.

### Statistics.

Two-way ANOVA was used to evaluate the effects of the interventions in the OB-PWL and OB-WM groups, the OB-RYGB and OB-LAGB groups, and the T2D-RYGB and T2D-LCD groups on body weight and metabolic variables. The effect of weight loss on β cell function was evaluated by using a mixed-effects regression model with (a) ISR as the dependent variable; (b) group, time during the glucose/meal ingestion test, and testing visit (before versus after weight loss) as fixed factors; and (c) plasma glucose concentration as a covariate. *P* ≤ 0.05 was considered statistically significant. Data are presented as mean ± SEM, unless noted otherwise.

### Study approval.

The study protocols were approved by the IRB at Washington University in St. Louis. All participants provided written informed consent before participating in this study.

## Author contributions

BM and SK designed the study. JCE performed the bariatric surgeries. BM, BWP, FM, MY, DPB, JCE, and SK and contributed to data acquisition, data analysis, and data interpretation. BM wrote the first draft of the paper. All authors contributed to the revision of the paper. BM and SK are the guarantors of this work, had full access to all the data in the study, and assume full responsibility for the integrity of the data and the accuracy of the data analysis.

## Supplementary Material

Supplemental data

ICMJE disclosure forms

## Figures and Tables

**Table 1:**
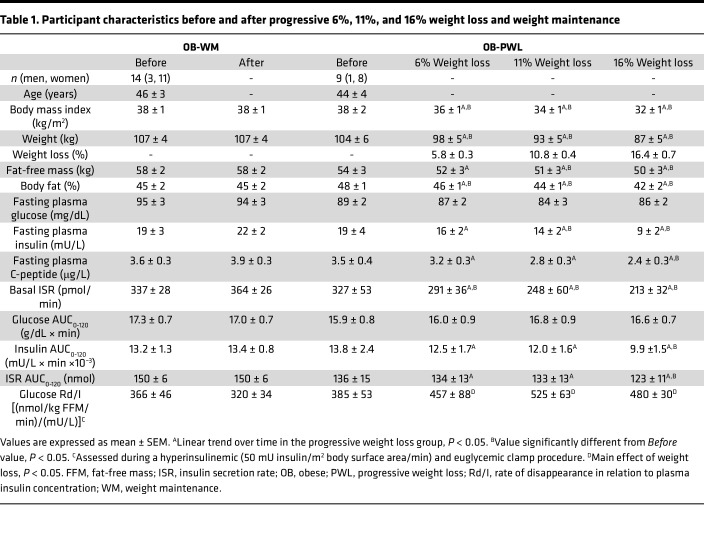
Participant characteristics before and after progressive 6%, 11%, and 16% weight loss and weight maintenance

**Table 2:**
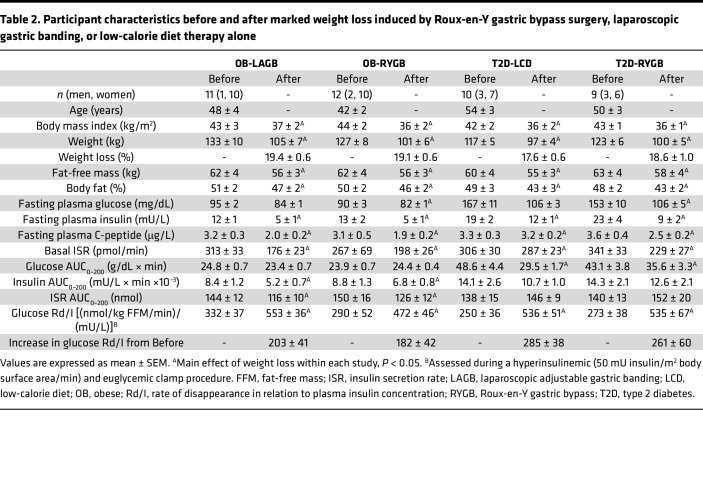
Participant characteristics before and after marked weight loss induced by Roux-en-Y gastric bypass surgery, laparoscopic gastric banding, or low-calorie diet therapy alone

## Version 1 05/11/2023

In-Press Preview

## Version 2 06/22/2023

Electronic publication
