# Supplementary material for: β Cell function after Roux-en-Y gastric bypass surgery or reduced energy intake alone in people with obesity
Source: JCI Insight. 2023 Jun 22;8(12):e170307. doi: 10.1172/jci.insight.170307 (PMC10371232; doi:10.1172/jci.insight.170307)
Supplement: Supplemental data [file jciinsight-8-170307-s005.pdf]

Online Supplemental Material

Beta-cell function after Roux-en-Y gastric bypass or reduced energy intake alone in people with obesity

Bettina Mittendorfer, Bruce W. Patterson, Faidon Magkos, Mihoko Yoshino, David P. Bradley, J. Cristopher Eagon, Samuel Klein

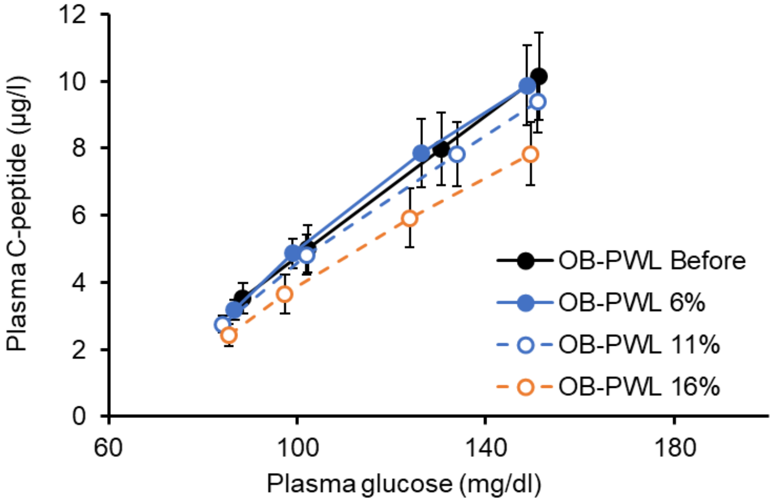

Supplemental Figure 1. Relationship between plasma C-peptide concentration and plasma glucose concentration before and during the first 30 minutes after ingesting 75 grams of glucose before and after progressive 6%, 11%, and 16% weight loss induced by a low-calorie diet. *Abbreviations*: OB, obese; PWL, progressive weight loss. Data are expressed as mean ± SEM; n = 9.
